# Intervention skills – a neglected field of research in medical education and beyond

**DOI:** 10.3205/zma001703

**Published:** 2024-09-16

**Authors:** Constanze Richters, Ralf Schmidmaier, Vitaliy Popov, Johann Schredelseker, Frank Fischer, Martin R. Fischer

**Affiliations:** 1 LMU Munich, LMU University Hospital, Institute of Medical Education, Munich, Germany; 2 LMU Munich, LMU University Hospital, Department of Medicine IV, Munich, Germany; 3 University of Michigan Medical School, Department of Learning Health Sciences, Ann Arbor, Michigan, USA; 4 University of Michigan, School of Information, Ann Arbor, Michigan, USA; 5 LMU Munich, Faculty of Medicine, Walther Straub Institute of Pharmacology and Toxicology, Munich, Germany; 6 LMU Munich, Department of Psychology, Munich, Germany

**Keywords:** intervention reasoning, cognitive intervention processes, clinical reasoning, health education

## Abstract

*Intervention* reasoning as a critical component of clinical reasoning has been understudied in medical education in contrast to the well-established field of diagnostic reasoning. This resonates in a lack of comprehensive understanding of the cognitive processes involved and a deficit in research to promote intervention skills in future clinicians. In this commentary, we present a conceptual framework for intervention reasoning that includes four phases: generating, selecting, implementing, and evaluating interventions. The conceptualization highlights cognitive processes such as developing interventions based on a patient’s diagnosis and signs and symptoms; selecting the most appropriate option by contrasting, prioritizing, and evaluating interventions in terms of feasibility, effectiveness, and the patient’s context-specific needs; and predicting patient outcomes within so-called “developmental corridors” to adjust treatments accordingly. In addition to these cognitive processes, interventions require collaborative activities, such as sharing information with other care providers, distributing roles among care teams, or acting together. Future research should validate the proposed framework, examine the impact of intervention reasoning on clinical outcomes, and identify effective training methods (e.g., simulation and AI-based approaches). In addition, it would be valuable to explore the transferability and generalizability of the model to other areas of health education and contexts outside of health education.

## Abstract (de)

*Interventionelles* Denken als kritischer Bestandteil der klinischen Entscheidungsfindung wurde in der medizinischen Ausbildung im Vergleich zur diagnostischen Entscheidungsfindung bisher zu wenig untersucht. Dies spiegelt sich in einem mangelnden Verständnis der kognitiven Prozesse und einem Forschungsdefizit zur Förderung der Interventionsfähigkeiten zukünftiger Ärztinnen und Ärzte wider. In diesem Kommentar stellen wir ein Konzept des interventionellen Denkens vor, das vier Phasen umfasst: Generierung, Auswahl, Implementierung und Evaluation von Interventionen. Das Konzept betont kognitive Prozesse wie die Entwicklung von Interventionen auf der Grundlage der Symptome und Diagnosen eines Patienten: Auswahl von Interventionen durch Vergleichen, Priorisieren und Bewerten von Interventionen hinsichtlich Machbarkeit, Effektivität und kontextspezifischen Bedürfnissen sowie die Vorhersage von Gesundheitsergebnissen innerhalb sogenannter „Entwicklungskorridore“, um Behandlungen entsprechend anzupassen. Neben diesen kognitiven Prozessen erfordern Interventionen auch kollaborative Aktivitäten wie den Informationsaustausch und die Verteilung von Rollen innerhalb des Betreuungsteams oder die gemeinsame Ausführung von Interventionsmaßnahmen. Zukünftige Forschung sollte das vorgeschlagene Konzept validieren, den Einfluss des interventionellen Denkens auf klinische Ergebnisse untersuchen und effektive Trainingsmethoden (z.B. Simulationen und KI-Techniken) identifizieren. Zudem ist es von Interesse, die Übertragbarkeit und Generalisierbarkeit des Modells auf andere Bereiche innerhalb und außerhalb der Gesundheitsbildung zu erforschen.

## Introduction

A fundamental aspect of clinical practice is the ability of clinicians to gather and integrate information about their patients, thereby reducing uncertainty regarding the etiology of symptoms and other phenomena – a process known as *diagnostic reasoning*. Once sufficient certainty has been achieved, this gathered information is used by clinicians to make decisions about the necessity and effectiveness of potential interventions, such as making decisions about medical drug prescriptions or surgical procedures, referred to as* intervention reasoning*. Diagnostic reasoning, along with intervention reasoning, can be summarized as clinical reasoning. 

The field of diagnostic reasoning has been the subject of considerable research [17], [24] and has recently attracted increasing attention beyond medical education [15]. The body of research has developed through studies that build on each other, thereby creating a robust understanding of how clinicians make accurate diagnoses [27]. In contrast, existing literature on intervention reasoning is rather limited and often lacks this cumulative and iterative development [10], resulting in a fragmented understanding of cognitive processes involved in intervention reasoning [8]. This is a concerning issue, as harmful interventions have a direct impact on patients’ lives [23]. 

One reason for the current limitations in research on intervention reasoning can be found in the primary focus of undergraduate medical education on diagnostic reasoning [22]. This focus is particularly relevant because the majority of preventable harm in clinical settings worldwide can be attributed to diagnostic errors [18]. However, our limited understanding of intervention reasoning further exacerbates the problem, potentially compromising patient outcomes even when diagnoses are accurate, such as fatal adverse drug reactions – a direct consequence of a patient treatment and a highly underestimated problem in clinical practice worldwide [23]. To prevent such intervention errors, a number of medical societies have developed a wide range of sophisticated consensus-based guidelines which include recommendations relating to both diagnostic and intervention (e.g., [2]). In addition, safety checks are an integral part of clinical practice, including clinical decision support systems [25] and medication safety checks (e.g., [6]). However, it must be acknowledged that such clinical guidelines and cognitive aids are not sufficient: A more comprehensive understanding of the development of intervention reasoning skills is required. 

For over a decade, pharmacologists and pharmacists have been advocating for the integration of intervention reasoning in drug prescribing into undergraduate medical curricula. Such integration would enhance interdisciplinary comprehension of the intervention process and facilitate the learning of related skills [22]. However, despite this call to action, structured intervention reasoning training remains absent from undergraduate medical education. Instead, intervention reasoning is currently dispersed across residency training (postgraduate medical education), with varying degrees of integration across medical subspecialties (e.g., internal medicine and dermatology). 

As the processes and activities involved in intervention reasoning may differ greatly from those involved in diagnostic reasoning, different educational and assessment approaches are required [7]. In addition to cognitive skills, intervention reasoning also involves interdisciplinary and interprofessional skills, as interventions frequently entail a high degree of interdisciplinary collaboration (e.g., radiology and internal medicine) and interprofessional teamwork (e.g., physiotherapy, occupational therapy, or pharmacy) in the delivery of clinical care [13]. 

In this commentary, we propose a conceptualization of intervention reasoning skills that builds on previous research. Given the limited existing literature, we propose which cognitive activities might be involved. This conceptual framework provides a foundation for further research and development of these important skills in medical education and potentially beyond, ultimately improving professional practice and outcomes for patients or other target groups. 

## Current state of research on intervention reasoning

In the context of complex problem solving, the term “diagnosing” is used to describe the process of identifying the problem and its underlying causes [1]. The term “intervening” is used to describe the act of solving and improving the identified problem. On the one hand, diagnoses serve as decision points for interventions [15]. On the other hand, intervention decisions serve to confirm or reject (working) diagnoses, such as when the patient’s response to an intervention generates new evidence that is pertinent to a specific suspected diagnosis [8]. This cyclical nature of diagnosis and intervention is particularly evident in emergency or high-acuity situations, where rapid and accurate assessment and treatment are required. In such situations, there is often a necessity for iterative cycles of diagnosis refinement and intervention adjustment (e.g., [19]). 

Existing research on intervention reasoning primarily originates from the healthcare field. Such studies use various terms, including therapeutic reasoning, management reasoning, and treatment decision making, in addition to intervention reasoning [10], [20], [28]. These terms are essentially synonymous with the concept of intervention reasoning. We use the term “intervention reasoning” in our conceptualization with the objective of potentially extending its application beyond healthcare to other fields. 

Three decades ago, the World Health Organization (WHO) developed a normative model for intervention reasoning that describes six steps in the intervention reasoning process for drug administration. These steps are based on the principles of “rational prescribing” [9]. The development of this manual was an early effort to establish intervention reasoning in undergraduate medical education. The six steps are as follows:

1. define the patient’s problem,
2. specify the therapeutic objective,
3. choose your treatment and verify its suitability,
4. start the treatment,
5. give information, instructions, and warnings, and
6. monitor the treatment and determine whether to stop it.

However, this model is constrained to drug prescriptions as interventions and lacks comprehensive descriptions of reasoning processes. Subsequent research from clinical pharmacology [22] has addressed this limitation by integrating the drug prescription model with theories from cognitive psychology and therapeutic reasoning, yet still with a focus on drugs. A recent scoping review of existing empirical research identified thinking processes such as *analyzing, synthesizing*, and *evaluating* as fundamental to intervention reasoning in diverse healthcare fields [10]. In conceptual articles from the clinical reasoning perspective, the *negotiation* of an action plan and the ongoing *monitoring* and *adjustment* of that plan are emphasized as components of intervention reasoning that involve different cognitive processes [7]. These cognitive processes include activities such as *contrasting, prioritizing*, and *selecting *from a range of feasible (justifiable) intervention options [8]. Moreover, Parsons et al. [20] introduced the concept of management scripts to describe and explain the organization of intervention options and decisions. *Management scripts* are mental schemas that help clinicians make decisions by providing structured, pre-compiled, conceptual knowledge for addressing health problems. They can be taught using templates that outline potential actions, which facilitates both the mastery of intervention reasoning and its teaching. Over time, such mental schemas are acquired through study and experience and activated or triggered in case- or problem-specific contexts. Due to the inherently complex nature of intervention in practice, clinicians must constantly adapt their management scripts to successfully treat patients, considering individual and contextual factors such as patients’ preferences, beliefs, expectations, and background. With increasing expertise, clinicians are increasingly able to adapt their management scripts for particular problems to the specific circumstances of a situation.

Based on a synthesis of these theoretical considerations and empirical findings, the following section describes the intervention process, and the activities involved. First, a definition is provided, and then a conceptualization is introduced, with detailed explanations of the components of intervention reasoning and related cognitive activities. The conceptualization encompasses a series of comprehensive, potentially generalizable intervention activities that can be employed not only for the purpose of intervention reasoning regarding the use of medication, but also for a multitude of other interventions.

## Proposing a conceptualization of intervention reasoning skills

**Figure 1:**
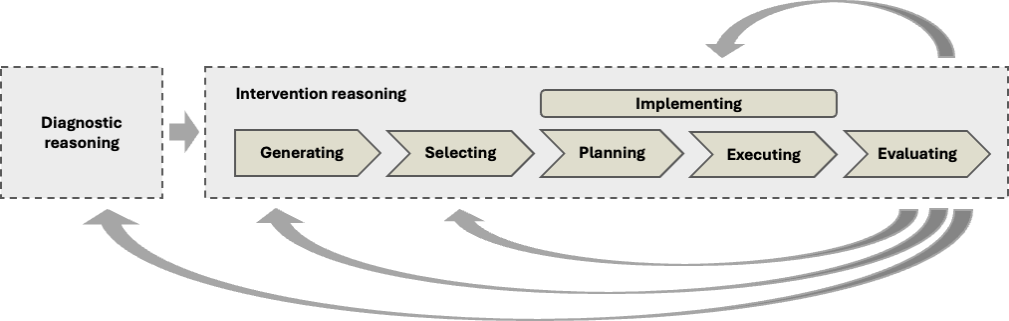
Working model of the conceptualization of intervention reasoning in context with diagnostic reasoning

Based on previous literature on clinical reasoning (references), we define intervention reasoning as the systematic process of generating, selecting, implementing, and continuously evaluating measures aimed at positively changing conditions or processes for patients. This process may involve individual as well as collaborative efforts, wherein argumentative communication about the suitability and efficacy of the selected interventions (e.g., presenting evidence, weighing alternatives, and reaching consensus) becomes paramount. Intervention measures can be considered both covet and overt actions in which clinicians engage in cognitive and collaborative endeavors (i.e., interdisciplinary or interprofessional) [26]. As the application of professional knowledge (e.g., clinical knowledge) to diagnostic activities is considered a crucial factor in the development of diagnostic reasoning skills [15], we similarly posit that the application of specific knowledge from distinct domains to intervention activities is a crucial factor in the development of intervention reasoning skills. Thus, intervention reasoning skills refer to the ability of clinicians to address problems by applying different types of knowledge, including professional knowledge (i.e., conceptual and strategic knowledge), knowledge of the consequences of interventions, and interprofessional knowledge, to intervention activities, according to professional standards. In medicine, professional knowledge refers to biomedical and clinical knowledge. This knowledge application entails considering patients’ individual characteristics, tolerating uncertainty and ambiguity, accepting multiple solutions, and complexity, monitoring treatment response and recognizing deviations from therapeutic goals, and considering contextual constraints [7]. Below, we describe the intervention process and activities (see figure 1 (Fig. 1)) in more detail: 

*Generating intervention options* involves the development of a range of solutions or strategies that are deemed reasonable and appropriate for addressing a specific problem or achieving a desired outcome. This activity requires a previous definition of the patient’s problem, as well as the specification of the intervention objective [9]. The exact causes of the problem may be known to a greater or lesser degree (i.e., the specificity of problem labeling or the accuracy of a diagnosis). For example, an appropriate treatment for a patient with an HIV infection (for which a specific diagnosis is available) would be antiretroviral therapy, a combination of drugs that suppress HIV replication. In practice, such a specific diagnosis is not always available at the time of initial treatment. For example, a patient may present with shortness of breath, which could be caused by a variety of conditions, including lung problems such as asthma, or heart problems such as a heart attack, allergies, or pulmonary embolism. However, it should be noted that a specific diagnosis is not necessarily required for initial treatment [7]. Immediate steps can and must be taken to stabilize the patient, including the administration of oxygen, monitoring of vital signs, application of anticoagulative treatment, and the provision of supportive care. These measures are critical to prevent the patient’s condition from worsening while diagnostic tests are performed to determine the underlying problem. Considering multiple potential causes and appropriate intervention options ensures a comprehensive approach to diagnosing and treating the patient. 

*Selecting interventions* involves *contrasting, prioritizing* and *evaluating* interventions based on the most appropriate, reasonable and justified strategies in terms of feasibility, effectiveness, and appropriateness to the specific context, acuity, complexity, and individual needs [7], [20]. For example, oncologists compare the potential benefits and risks of chemotherapy, radiation therapy, and surgery and evaluate them in terms of their effectiveness in shrinking tumors, managing symptoms, and improving survival rates (contrasting). Oncologists also rank these different intervention options based on criteria such as urgency or effectiveness, for example, prioritizing the control of cancer symptoms over the actual treatment of the cancer (prioritizing). In selecting appropriate treatments, clinicians also consider how well the treatment meets the patient’s individual needs and preferences, taking into account factors such as treatment side effects and the general condition of the patient (*Threshold to Treat*; [21]), recovery time, and long-term outcomes (evaluating). Thus, selecting interventions involves not only selecting a standard treatment for a specific problem, but also the suitability to that standard treatment for the individual patient [9]. For example, in the case of a patient diagnosed with hypertension, the initial treatment option is typically Lisinopril. However, if the patient has a medical history of angioedema, this first-line strategy is contraindicated. Instead of Lisinopril, amlodipine, a calcium channel blocker, is prescribed to reduce the risk of angioedema. The result of the selection process may be a sequential use of interventions or a combination of several interventions at the same time in case the patient presents with health problems that can be attributed to more than one cause. This is also known as dual diagnosis [16], when a complex condition requires an integrated treatment approach that addresses both a primary disorder and a co-morbidity concurrently. For example, this could include a patient with major depressive disorder and alcohol dependence, or a patient with HIV who develops Pneumocystis jirovecii pneumonia (PCP) as an opportunistic infection. The simultaneous treatment of both disorders (e.g., mental health and substance use disorders) is imperative, as each condition can exacerbate the other.

*Implementing interventions* involves

1. planning and
2. executing interventions.

In the process of *planning* interventions, clinicians define the goals and rules that will be used for monitoring and adjusting the intervention as it is implemented. The planning process also involves sequencing interventions based on their (interactive) effectiveness. For example, a specific drug, such as capecitabine, may enhance the effectiveness of radiation therapy; chemotherapy may be prioritized to reduce the size of the tumor, followed by surgery to remove any remaining cancerous tissue. The rules, along with the previously selected and sequenced interventions, are recorded in an implementation plan, which ensures a transparent exchange of information with the patient, family and all care providers involved in the intervention over time [9]. The implementation plan comprises a monitoring and adjustment plan [7]. The components of the *monitoring plan* include, among others, establishing the goal of the intervention, specifying monitoring parameters (e.g., clinical symptoms), establishing monitoring frequency (e.g., weekly or monthly), and establishing thresholds for adjustment (e.g., patient’s symptoms worsen or improve). The *adjustment plan* includes specific decision criteria and options for adjustments based on those decision criteria. These may include changing a patient’s medication dosage, switching to a different medication, changing the frequency of administration, or adding additional therapies. To illustrate, if a patient’s blood pressure remains high despite taking 10 mg of lisinopril daily, the adjustment plan may include increasing the dosage to 20 mg daily.

The cognitive processes that enable the specifications and the development of the intervention plan is what we call the “prediction of developmental corridors”. The term “developmental corridor” describes the typical range of variation in a patient’s health status, which can be influenced by several factors, including social, individual, genetic, and interventional factors. This range includes potential changes when interventions are effective. However, it is not uncommon for a patient’s condition to deviate from these established boundaries. Therefore, predictions for developmental corridors consider outcomes both within and beyond this range. Moreover, the predictions in the plan facilitate the transparent delineation of responsibilities and facilitate the transfer of those responsibilities to the patient or to third parties such as other physicians, caregivers, or family members. To the best of our knowledge, the cognitive process of predicting intervention-based developmental corridors is largely unexplored. As a result, there is a lack of knowledge regarding the optimal teaching and support strategies for such prediction processes. 

After the planning phase, the intervention(s) are executed. While the intervention plan is primarily a (collaborative) cognitive process, executing the plan involves practical issues such as organization, manual skills, and communication. An example is the performance of surgery, wherein surgeons and anesthetists are required to coordinate the team actions of multiple health professionals and equipment (e.g., assigning roles and tasks), perform the surgery using surgical and anesthesiological skills (e.g., inducing and supervising anesthesia, incision and drainage of an abscess, suturing and knot tying), and communicate effectively with team members (e.g., using techniques such as closed-loop communication). 

*Evaluating interventions* refers to the assessment of the intervention by clinicians in terms of its efficacy in addressing the specific problem or achieving the previously established goal. Evaluation is closely linked to monitoring. These processes can result in two primary outcomes: no adjustment or an adjustment. For example, if clinicians decide not to adjust the current intervention based on their monitoring, they may have determined that the current treatment aligns with the predictions of the developmental corridors. Alternatively, particularly in the final evaluation of an intervention, clinicians may conclude that the patient’s disease has been effectively cured (e.g., a patient’s bacterial pneumonia has been successfully treated with antibiotics). If clinicians opt to adjust the current intervention, they may decide that the implementation needs to be refined. This may entail adapting the intervention itself (e.g., changing the sequence of different treatments or adjusting the drug dosage) or optimizing the execution of the intervention (e.g., better communication with the individual or improving contextual factors). In addition, clinicians may opt to generate or select novel intervention options to complement of supplant current ones. For example, antidepressants may be added to existing behavioral therapy to treat a patient with moderate depression, or an antibiotic may be substituted for another because it is not effective in treating a patient with PCP. Finally, clinicians may revert to diagnostic reasoning (e.g., deciding for further testing based on the *Threshold to Test*; [21]). This process of diagnostic reasoning is continued as long as sufficient certainty for further interventional decisions has been reached.

## Learning how to intervene: Identifying conditions for effective training of intervention reasoning

Considering our conceptualization of intervention reasoning skills, we propose some concrete research questions for future investigation. First, the conceptualization of intervention reasoning must be validated. Future research would benefit greatly from studies that contribute to different aspects of validity. Therefore, we call for validation studies, such as observational or interview studies, which test and advance the preliminary conceptualization presented in this article in various ways. With regard to construct validity, the following questions could be addressed:

1. To what extent are the intervention activities outlined in the conceptualization used by clinicians?
2. In what ways do more vs. less experienced clinicians follow the proposed sequence of activities in a linear fashion, or do they engage in a more nonlinear, dynamic process of intervention reasoning? We hypothesize that while experienced clinicians may not consciously follow a linear sequence, their cognitive processes likely still encompass all the proposed activities, albeit in a more fluid and interconnected manner.
3. Does intervention reasoning refer to a single skill (i.e., all sub-dimensions are correlated) or to a set of skills (i.e., different sub-skills explain variance independently)? We hypothesize that intervention reasoning skills refer to a set of skills rather than a single skill.

In terms of predictive and content validity, the following questions could be addressed:

1. To what extent is professional knowledge (i.e., conceptual and strategic knowledge) correlated with successful engagement in intervention activities and overall intervention success?
2. To what extent can the engagement in either single intervention activities or multiple intervention activities be used to predict the success of interventions (i.e., improvement in a patient’s health status)?

With regard to joint intervening, the following question can be addressed: To what extent can social skills and knowledge in the collaborator’s domain be identified as factors that contribute to successful engagement in interdisciplinary or interprofessional intervention activities?

After advancing the understanding of intervention reasoning as a construct, research could address the identification of conditions for effective training of intervention reasoning skills. A highly promising educational approach is *simulation-based learning*, which has been demonstrated to be an effective approach for developing complex skills such as (collaborative) diagnostic reasoning in medicine and beyond [5]. The effectiveness of simulations for learning can be enhanced by integrating additional scaffolding for the learning process, such as reflection prompts, external collaboration scripts or worked examples [5]. Educational researchers have recently introduced the concept of *representational scaffolding* [11], which relates to features that are closely aligned with the demands of professional practice. These features include informational complexity (i.e., the amount and degree of interconnectedness of information and the prominence of cues), situational dynamics (i.e., changes in the practice situation over time), and agency/responsibility (i.e., the demands on a professional’s ability to act flexibly and appropriately). The effectiveness of this type of scaffolding in learning intervention reasoning, has yet not been empirically investigated. In the following, we propose a set of research questions that must be addressed to advance our understanding of how intervention reasoning skills can be fostered in medical education and beyond.

1. To what extend can the teaching concepts established for diagnostic reasoning be applied to the teaching of intervention reasoning? Where do they differ?
2. To what extent can intervention reasoning skills be enhanced by simulation-based training (e.g., simulated patient cases)?
3. Is it feasible to foster intervention reasoning skills using the same simulations employed for diagnostic reasoning training?
4. To what extent do simulations need to be simplified in terms of complexity, dynamics, and agency/responsibility to facilitate the learning of intervention reasoning skills?
5. How can problem-based learning and inquiry learning be complemented with direct instruction to effectively foster intervention reasoning skills?
6. How can digital learning environments be used to foster intervention reasoning skills, and what is an effective role of AI in personalizing the learning experience?
7. What is the impact of the transfer of intervention reasoning skills from educational settings to clinical practice on the rate of diagnostic errors and subsequent patient outcomes, particularly in the context of high-risk conditions (e.g., stroke, sepsis, pneumonia, lung cancer)?

Finally, as previously stated at the outset of this commentary, an open question is the extent to which intervention reasoning skills are domain specific, or whether such skills are, in terms of problem solving and decision-making processes, generalizable across similar domains in medicine and health education. In addition, basic research on learning to intervene would greatly benefit from studies that address the generalizability of these findings to contexts also outside of medical and health education. Researchers from a variety of fields, including education, psychology, and medicine, have recently emphasized the need for cross-disciplinary research on learning and teaching to address the complex issues of education in the 21^st^ century [14]. In this context, teacher education is particularly interesting, as the processes of diagnosis and intervention also play an important role in the professional practice of teachers. A fundamental responsibility of teachers is to assess students’ current learning status in relation to various factors such as cognitive misconceptions or, more broadly, gaps in knowledge, lack of skills, or motivation. Teachers then strive to provide optimal support for student’s learning, based on the student’s identified needs. Previous research has examined the similarities between medical and teacher education, suggesting potential parallels in areas such as professional knowledge [12] and diagnostic activities (e.g., [3]). A cross-disciplinary research perspective on intervention reasoning skills, through the collaboration between psychology and other fields, has the potential to assist us in leveraging the insights generated from medical education to gain general knowledge outside of medicine.

The conceptualization of intervention reasoning presented in this commentary serves as a foundation for advancing our understanding of these skills, which are crucial for professional practice in healthcare and beyond. 

## Authors’ ORCIDs

- Constanze Richters: [0000-0003-1593-3543]
- Ralf Schmidmaier: [0000-0003-3541-3588]
- Vitaliy Popov: [0000-0003-2348-5285]
- Johann Schredelseker: [0000-0002-6657-0466]
- Frank Fischer: [0000-0003-0253-659X]
- Martin R. Fischer: [0000-0002-5299-5025]

## Competing interests

The authors declare that they have no competing interests.
